# A new multitest correction (SGoF) that increases its statistical power when increasing the number of tests

**DOI:** 10.1186/1471-2105-10-209

**Published:** 2009-07-08

**Authors:** Antonio Carvajal-Rodríguez, Jacobo de Uña-Alvarez, Emilio Rolán-Alvarez

**Affiliations:** 1 Departamento de Bioquímica, Genética e Inmunología, Facultad de Biología, Universidad de Vigo, 36310, Vigo, Spain; 2 Departamento de Estadística e Investigación Operativa, Facultad de Ciencias Económicas y Empresariales, Universidad de Vigo, 36310, Vigo, Spain

## Abstract

**Background:**

The detection of true significant cases under multiple testing is becoming a fundamental issue when analyzing high-dimensional biological data. Unfortunately, known multitest adjustments reduce their statistical power as the number of tests increase. We propose a new multitest adjustment, based on a sequential goodness of fit metatest (SGoF), which increases its statistical power with the number of tests. The method is compared with Bonferroni and FDR-based alternatives by simulating a multitest context via two different kinds of tests: 1) one-sample t-test, and 2) homogeneity G-test.

**Results:**

It is shown that SGoF behaves especially well with small sample sizes when 1) the alternative hypothesis is weakly to moderately deviated from the null model, 2) there are widespread effects through the family of tests, and 3) the number of tests is large.

**Conclusion:**

Therefore, SGoF should become an important tool for multitest adjustment when working with high-dimensional biological data.

## Background

Statistical tests are a fundamental scientific tool for contrasting alternative hypotheses by rejecting or not the null one given an *a priori *fixed significance level. Such a methodology may have two types of associated errors: type I error, i.e. the rejection of the null hypothesis when it is true (a false discovery or false positive) and type II error i.e. the acceptance of the null hypothesis when the alternative one is true (a false negative [1]). Most statistical tests traditionally aim to control type I error. However, such a strategy was originally developed to test a single null hypothesis, and an undesirable high rate of false discoveries may be obtained when working with families of comparisons under simultaneous consideration. Different strategies have been considered to deal with this problem. The control of the familywise error rate (FWER; [2]) is performed by Bonferroni likewise techniques. The aim of the FWER is to control the probability of making one or more type I errors in families of simultaneous comparisons. Alternatively, false discovery rate (FDR) based methods aim to control the proportion of false discoveries among the total ones (i.e. the proportion of the rejected null hypotheses which are erroneously rejected [3-5]). Multitest adjustment strategies have gained attention since the apparition of the so-called high-dimensional biological data as a consequence of the 'omic' technologies. Therefore, in some research areas the number of tests accomplished has increased dramatically due to the recent technological improvements [6-9]. For example, in genomic and microarray studies it is becoming common to work simultaneously with more than 20,000 tests [6], and future studies may produce analysis of complete genomes with many thousands of polymorphisms between a few species [10].

Thus, there is an obvious interest to know which of the available multitest adjustments is the most useful. Benjamini and Hochberg [3] demonstrated that the direct control of FDR increases considerably the statistical power of multitest adjustment. This is expected because any procedure that controls the FWER also controls the FDR being therefore more stringent than the FDR-based methods [3]. Since then, several variants of the FDR and Bonferroni adjustments have been proposed [11-19], although there is no clear consensus about which is the best multitest adjustment in all conditions [11]. In any case, all available multitest adjustments show the inconvenience of decreasing statistical power when increasing the number of tests [16]. This occurs because all methods adjust each single test error rate according to the number of tests used. The consequence of this adjustment is that the higher the number of comparisons the lower the chance to detect even one significant (true discovery) case using any multitest correction. Such a conservative control of type I error is often not very useful from an experimentalist point of view [16]. In addition, multitest adjustment needs accurate estimates of the *p*-values [17,20,21]. For example, we need 5 decimal digits to use the Bonferroni multitest adjustment with 1000 tests at a significance level of 5% (α_adjusted _= 0.00005). Furthermore, the use of non-parametric (ranked) methods at very small sample sizes (for example using 3 replicates; see [5]) may produce inaccurate probabilities, which makes less effective the multitest adjustment. In such cases, it may be suggested the use of their parametric counterparts, but if the parametric assumptions are not met, this may produce biased probabilities which will be useless under any multitest method [1]. Due to these and similar problems some authors have been reluctant to use multiple test adjustments indiscriminately [22], or even recommend that multiple adjustments should not be used [23].

Ideally, any multitest correction should show a large statistical power and a small FDR under a small number of comparisons, and its statistical power should increase when increasing the number of tests, as most statistical tests do in relation to sample size. As explained above this is not the situation with any of the available multitest adjustments. Here, we propose a new multitest adjustment methodology based on a sequential goodness of fit (SGoF) metatest. This method will help the researcher to decide which of the tests, previously ranked based on their *p*-values, would be true discoveries. As desired, SGoF increases its statistical power when the number of tests increases. In the present work we formalize the method, giving power and type I error expectations. We also perform simulations both via multiple one-sample t and homogeneity tests, to compare SGoF with three alternative multitest adjustment methods: Bonferroni (B), Sequential Bonferroni (SB) and the Benjamini and Hochberg [3] (BH) which is the original implementation to control for FDR.

Our results show that SGoF can be a valuable approach for multitest adjustments with high-dimensional biological data. Under the most favorable conditions (large number of tests, weak to medium deviations from the null model, and a relatively high proportion of such deviations or effects) this test can show a statistical power up to two orders of magnitude higher than the BH and Bonferroni methods without increasing appreciably the false discovery rate (FDR).

## Results

### Definition of Sequential Goodness of Fit (SGoF) metatest

Consider testing a set of *S *independent comparisons at significance level α, with their respective null hypotheses *H*_1_,*H*_2_, ..., *H*_*S*_. Let *P*_1 _≤ *P*_2 _≤...≤ *P*_*S *_be the ranked *p*-values associated to each test, and denote by *H*_*i *_the null hypothesis corresponding to *P*_*i*_. Let *K *be the observed number of rejections after performing the *S *tests individually at level α. Provided that the *S *nulls are true the expected number of rejections (i.e. false discoveries) is *S *× α. Hence, the observed value *K *can be compared to the expectation in order to reach a conclusion about its significance; that is, to check whether the amount of significant tests could be explained by chance. The SGoF metatest performs a goodness-of-fit (again at level α) test of one degree of freedom comparing the observed (*K*) and the expected (*S *× α) numbers of rejections on the family of tests accomplished. This goodness-of-fit metatest is defined as an exact binomial test. However, when *S *≥ 100 it is approximated by a chi-squared or a G-test, both of them approximating a chi-squared distribution with one degree of freedom (see Methods for a detailed description of the algorithm). Let *k*_α _be the critical value, given *S *and α, for such metatest; that is, a rejection at level α occurs when *K *≥ *k*_α_. Thus, *k*_α _is the 1-α percentile of the binomial distribution (1-α percentile of the χ^2 ^distribution with 1 degree of freedom if a chi-squared or a G-test statistic is used). Here, "rejection" means that at least one of the null hypotheses is false. More specifically, in the case of rejection, the SGoF metatest concludes that the *K*-*k*_α_+1 hypotheses with the smallest *p*-values (these are, *H*_1_, *H*_2_,..., *H*_*K*-*kα*+1_) are false. Clearly, this is a proper subset (typically much smaller) of the initial set of *K *rejected hypotheses when performing the individual testing. As we state below, this procedure controls for FWER.

Consider the following example of application (also see the Algorithm section in Methods). Imagine that *S *= 10,000 tests are performed and *K *= 600 are significant at α = 0.05. In the case of the exact binomial test, the critical value corresponding to significance level α = 0.05 and *S *= 10,000 tests is *k*_α _= 536, which corresponds to the 95% percentile of the Binomial (10000, 0.05) distribution. Therefore, if we have an observed value of *K *= 600 this means that the 600-536+1 = 65 hypotheses with the smallest *p*-values will be considered significant.

Note that, unlike the Benjamini and Hochberg FDR controlling procedure [3], the proposed SGoF test does not decide which hypotheses are false by comparing the attained *p*-values to some values of reference. Rather, the question that is addressed by the SGoF statistic is: are there too many rejections (when testing individually the *S *hypotheses) with respect to the expected amount of them? How many among these rejections are not attributable to chance?

Now we describe some basic properties of the SGoF metatest. Property 1 implies that SGoF controls for the familywise error rate (FWER) in the weak sense. Property 2 evaluates the per comparison error rate of SGoF. The error rates in Properties 1 and 2 are analyzed under the assumption that the *S *null hypotheses to be tested in a simultaneous way are true. Finally, we investigate in Property 3 the power of SGoF to reject at least one hypothesis in the case that a portion of nulls is false.

#### Property 1

SGoF metatest controls for FWER in the weak sense, that is, under the intersection null hypothesis (all nulls are true). This is an immediate consequence of its definition. Note that FWER is the probability of committing one or more than one type I error. In our case, this is the probability of *K *≥ *k*_α_, which, by definition of *k*_α_, is smaller than or equal to α. Recall that FWER and FDR coincide when the *S *nulls are true [3], so our method also controls directly for FDR in this situation.

#### Property 2

The per comparison error rate (PCER; [3]) of the SGoF test is

(1)

where *I*(*K *≥ *k*_α_) is the indicator of the event *K *≥ *k*_α_, having value 1 if the assert is true and 0 otherwise, which can be evaluated from the null distribution of *K*. Therefore, PCER reveals (on average) the probability of committing a type I error for each individual hypothesis (before the *p*-values are given). For example, if the number of hypotheses (*S*) is 10,000, and the significance level (*α*) is 5%, PCER(SGoF) approximately equals 5 × 10^-5 ^(approximation based on 500 samples of size 1000 from a binomial random variable). This is about ten times α/S, a fact that explains the higher power of SGoF when compared with other FWER tests as the classical Bonferroni one (see below). By simulating several values of *S*, we have estimated that PCER(SGoF) ≈ 4α/*S *for *S *= 10^3^, ≈ 10α/*S *for *S *= 10^4^, ≈ 29α/*S *for *S *= 10^5^, and ≈92α/*S *for *S *= 10^6 ^(in the case that α = 0.05). Therefore, it seems that the SGoF per test error rate is proportional to α/S by a factor that increases with the number of tests, *S*, resolving in this way the trade-off between type I error and statistical power. This means that the higher *S *the higher the probability that the metatest rejects each null hypothesis (relative to that of Bonferroni).

#### Property 3

The probability that SGoF rejects at least one null hypothesis steadily increases up to one as *S *increases, provided that a given portion of the null hypotheses remains false. To illustrate this property, assume that there is a proportion λ = *S*_0_/*S *(λ < 1) of true null hypotheses among the *S *being tested, and that the individual probability of rejection at level α of the 1-λ false hypotheses is α_1 _rather than α (with α_1 _> α). Then, the probability that SGoF rejects one or more than one hypotheses is approximately given by the probability that a standard normal is less than the critical value

(2)

where z_α _is the (1-α)th percentile of the standard normal. Note that z_β _is the (1-β) percentile and 1-β is the power of the test which in the case of SGoF, means the power to reject that all nulls are true (intersection null hypothesis) i.e. to detect that at least one null is false. This critical value *z*_β _approaches to infinity as S increases. Therefore, the power to detect that at least one null is false increases with *S*. Note that the factor α_1_-α controls the closeness of the alternative hypothesis to the null, so (as one can expect) the power of SGoF decreases for close alternatives (weak effects). As it is known to occur with other adjustment methods [3].

### Simulations

Simulations were run under two different scenarios: 1) the null model is always true (intersection null hypothesis), and 2) the alternative model is true in some of the *S *tests (see Methods section).

#### Null model is always true

Expected and detected numbers of false positives were compared under the simulation design (Table 1). The results were similar for both one-sample t and G homogeneity tests (see Methods section). The mean percentage of false positives (*S*_α_) obtained in the simulation was close to the theoretical expectation (α = 5%) in all cases. Only the results for SB, BH and SGoF are shown because the B method produced exactly the same values as SB. The mean and standard deviations of the detected significant cases are presented (in %) for 1000 replicates. Clearly, the three methods showed rather similar type I errors. SGoF had slightly higher variability through replicates but smaller across the set of cases simulated (Table 1). In any case, all multitest methods maintained low levels of false rejection rates when the null hypothesis was true.

**Table 1:** Mean percentage of significant cases detected when the null hypothesis was always true.

	*N*	*S*	*S*_α_	SB	BH	SGoF
t-test	5	100	4.95 ± 0.421	0.042 ± 0.0139	0.046 ± 0.0171	0.047 ± 0.0306
	5	1000	5.02 ± 0.237	0.006 ± 0.0016	0.007 ± 0.0020	0.008 ± 0.0265
	5	10000	5.00 ± 0.129	0.001 ± 0.0002	0.001 ± 0.0002	0.002 ± 0.0116
	10	100	4.97 ± 0.412	0.063 ± 0.0184	0.068 ± 0.0209	0.042 ± 0.0485
	10	1000	4.97 ± 0.236	0.005 ± 0.0015	0.005 ± 0.0016	0.007 ± 0.0275
	10	10000	5.00 ± 0.130	0.000 ± 0.0001	0.000 ± 0.0002	0.002 ± 0.0079
	20	100	5.03 ± 0.419	0.045 ± 0.0157	0.052 ± 0.0194	0.045 ± 0.0294
	20	1000	4.99 ± 0.233	0.006 ± 0.0021	0.006 ± 0.0025	0.004 ± 0.0076
	20	10000	5.02 ± 0.130	0.001 ± 0.0001	0.001 ± 0.0002	0.002 ± 0.0087
				
			Mean	0.019 ± 0.0242	0.021 ± 0.0267	0.018 ± 0.0204

	20	100	5.16 ± 0.421	0.049 ± 0.0149	0.052 ± 0.0166	0.046 ± 0.0356
	20	1000	5.21 ± 0.239	0.004 ± 0.0014	0.004 ± 0.0017	0.014 ± 0.0157
G-test	20	10000	5.22 ± 0.133	0.001 ± 0.0001	0.001 ± 0.0003	0.019 ± 0.0558
	40	100	5.05 ± 0.427	0.084 ± 0.0217	0.092 ± 0.0282	0.066 ± 0.0546
	40	1000	5.12 ± 0.239	0.003 ± 0.0014	0.004 ± 0.0021	0.008 ± 0.0108
	40	10000	5.09 ± 0.132	0.001 ± 0.0002	0.001 ± 0.0002	0.005 ± 0.0215
			Mean	0.024 ± 0.0350	0.026 ± 0.0381	0.026 ± 0.0243

Values are averages through 1,000 replicates and their ± standard deviations. The simulated null models were tested under t or homogeneity G tests (see text). *N*: sample size. *S*: number of tests. *S*_α_: Percentage of significant tests. SB: Sequential Bonferroni. BH: Benjamini and Hochberg. SGoF: Sequential Goodness of Fit.

#### Alternative models

We studied the ability of different multitest adjustments to detect significant cases when there is an increasing proportion of tests undergoing a true effect (% effect, see Methods). Different sample sizes were studied ranging from 5 to 20 for the one-sample t tests or from 20 to 40 for the homogeneity tests (see Methods). The results were similar for both kinds of tests. Again, the B method is not presented as it was nearly identical to the SB one. When the effect was weak (Tables 2 and 3) BH always shows an equal or higher mean statistical power than SB, although in both cases the detection of true discoveries is extremely poor. When the effect was strong (Tables 4 and 5), BH has high power only with the largest sample sizes. A quite important pattern can be followed from these tables. When the number *S *of tests increases then the power decreases for SB and BH but increases for SGoF. The latter occurs as predicted from *property *3 above.

**Table 2:** Percentages of significant cases detected (*Detected*) and false discovery rate (*FDR*) after multitest adjustment when the *p*-values come from families of one-sample t tests where some (% effect) of the alternative hypotheses were true.

	*Weak*			SB		BH		SGoF	
						
*N*	*% effect*	*S*	*Significant*	*Detected*	*FDR*	*Detected*	*FDR*	*Detected*	*FDR*
5	5%	100	5.2	0.04	98	0.04	98	0.07	85
5	5%	1000	5.2	0.01	78	0.01	80	0.01	91
5	5%	10000	5.2	0.00	91	0.00	88	0.02	89
5	10%	100	5.4	0.06	86	0.06	87	0.09	83
5	10%	1000	5.5	0.01	83	0.01	84	0.04	78
5	10%	10000	5.5	0.00	80	0.00	83	0.11	80
5	20%	100	5.9	0.08	57	0.08	60	0.12	64
5	20%	1000	6.0	0.01	57	0.01	58	0.13	63
5	20%	10000	6.0	0.00	70	0.00	69	0.54	63
				
			Mean	0.02	77.8	0.02	78.6	0.12	77.3
			SD	0.030	14.18	0.030	13.45	0.161	11.24

10	5%	100	5.7	0.05	72	0.06	70	0.09	80
10	5%	1000	5.6	0.01	69	0.01	67	0.06	77
10	5%	10000	5.6	0.00	71	0.00	71	0.22	74
10	10%	100	6.2	0.06	56	0.08	57	0.19	67
10	10%	1000	6.2	0.01	44	0.01	47	0.24	59
10	10%	10000	6.3	0.00	44	0.00	45	0.84	61
10	20%	100	7.6	0.12	35	0.15	35	0.52	42
10	20%	1000	7.5	0.01	30	0.02	30	1.14	42
10	20%	10000	7.5	0.00	27	0.00	30	2.11	44
				
			Mean	0.03	49.8	0.04	50.2	0.60	60.7
			SD	0.041	17.85	0.051	16.72	0.674	15.15

20	5%	100	6.4	0.14	33	0.17	35	0.20	60
20	5%	1000	6.5	0.02	31	0.03	30	0.33	51
20	5%	10000	6.4	0.00	18	0.00	19	1.01	53
20	10%	100	7.8	0.24	17	0.32	18	0.61	38
20	10%	1000	7.9	0.03	13	0.06	16	1.53	36
20	10%	10000	7.9	0.01	7	0.01	10	2.46	40
20	20%	100	10.7	0.36	8	0.57	10	2.24	24
20	20%	1000	10.8	0.06	6	0.15	9	4.38	25
20	20%	10000	10.8	0.01	4	0.04	6	5.35	27
				
			Mean	0.10	15.22	0.15	17	2.01	39.3
			SD	0.126	10.67	0.188	9.89	1.81	13.02

*Weak*: The alternative hypothesis is weak implying that data come from a Normal(0.36, 1). *N*: sample size. % *effect*: % of real true alternatives. *S*: number of tests. *Significant*: % of significants before adjustment. *Detected*: % of significant tests after adjustment. *FDR*: false discovery rate. SB: Sequential Bonferroni. BH: Benjamini and Hochberg. SGoF: Sequential Goodness of Fit.

**Table 3:** Percentages of significant cases detected (*Detected*) and false discovery rate (*FDR*) after multitest adjustment when the *p*-values come from families of homogeneity tests where some (% effect) of the alternative hypotheses were true.

	*Weak*			SB		BH		SGoF	
						
*N*	*% effect*	*S*	*Significant*	*Detected*	*FDR*	*Detected*	*FDR*	*Detected*	*FDR*
20	5%	100	5.7	0.10	73	0.10	72	0.10	77
20	5%	1000	5.7	0.01	53	0.01	57	0.07	78
20	5%	10000	5.7	0.00	65	0.00	61	0.25	55
20	10%	100	6.2	0.07	51	0.08	51	0.18	68
20	10%	1000	6.1	0.01	41	0.01	40	0.19	56
20	10%	10000	6.1	0.00	40	0.00	33	0.68	33
20	20%	100	6.8	0.14	39	0.16	38	0.30	52
20	20%	1000	7.0	0.01	24	0.01	25	0.70	35
20	20%	10000	7.0	0.00	16	0.00	16	1.58	19
				
			Mean	0.04	44.7	0.04	43.7	0.45	52.6
			SD	0.052	18.17	0.058	18.06	0.482	20.37

40	5%	100	6.0	0.14	45	0.15	46	0.13	69
40	5%	1000	6.0	0.02	31	0.02	33	0.15	57
40	5%	10000	6.0	0.00	22	0.01	23	0.61	37
40	10%	100	7.0	0.21	26	0.25	28	0.33	51
40	10%	1000	7.0	0.03	18	0.04	20	0.71	38
40	10%	10000	7.0	0.01	12	0.01	14	1.56	17
40	20%	100	8.9	0.34	20	0.45	21	1.02	32
40	20%	1000	8.9	0.05	8	0.10	9	2.50	21
40	20%	10000	8.9	0.01	5	0.04	7	3.44	6
				
			Mean	0.09	20.8	0.12	22.3	1.16	36.4
			SD	0.117	12.34	0.148	12.23	1.143	20.19

*Weak*: The alternative hypothesis is weak. *N*: sample size. % *effect*: % of real true alternatives. *S*: number of tests. *Significant*: % of significants before adjustment. *Detected*: % of significant tests after adjustment. *FDR*: false discovery rate. SB: Sequential Bonferroni. BH: Benjamini and Hochberg. SGoF: Sequential Goodness of Fit.

**Table 4:** Percentages of significant cases detected (*Detected*) and false discovery rate (*FDR*) after multitest adjustment when the *p*-values come from families of one-sample t tests where some (% effect) of the alternative hypotheses were true.

	*Strong*			SB		BH		SGoF	
						
*N*	*% effect*	*S*	*Significant*	*Detected*	*FDR*	*Detected*	*FDR*	*Detected*	*FDR*
5	5%	100	6.6	0.09	62	0.11	62	0.23	59
5	5%	1000	6.7	0.01	53	0.01	54	0.47	60
5	5%	10000	6.7	0.00	42	0.00	47	1.24	60
5	10%	100	8.5	0.14	31	0.17	30	0.85	46
5	10%	1000	8.4	0.01	36	0.02	35	1.97	43
5	10%	10000	8.3	0.00	31	0.00	30	2.88	45
5	20%	100	11.7	0.17	18	0.28	18	3.03	26
5	20%	1000	11.7	0.02	17	0.03	18	5.28	28
5	20%	10000	11.7	0.00	22	0.00	21	6.23	29
				
			Mean	0.05	34.7	0.07	35	2.46	44
			SD	0.067	15.48	0.099	16.04	2.118	13.91

10	5%	100	8.7	0.49	8	0.75	11	0.95	24
10	5%	1000	8.6	0.09	6	0.27	8	2.25	23
10	5%	10000	8.6	0.01	4	0.11	6	3.21	28
10	10%	100	12.3	1.00	5	2.08	8	3.56	14
10	10%	1000	12.3	0.18	2	1.18	6	5.92	17
10	10%	10000	12.3	0.03	2	0.93	5	6.86	20
10	20%	100	19.5	1.91	3	6.37	5	10.53	9
10	20%	1000	19.6	0.33	1	5.38	4	13.23	11
10	20%	10000	19.6	0.05	1	5.30	4	14.17	12
				
			Mean	0.45	3.6	2.49	6.3	6.74	17.6
			SD	0.631	2.40	2.481	2.29	4.856	6.58

20	5%	100	9.8	2.92	2	4.18	7	1.59	6
20	5%	1000	9.7	1.38	0	4.04	5	3.35	4
20	5%	10000	9.7	0.47	0	3.97	5	4.24	6
20	10%	100	14.4	5.86	1	9.06	5	5.49	2
20	10%	1000	14.3	2.75	0	8.90	5	7.93	3
20	10%	10000	14.3	0.95	0	8.85	4	8.90	5
20	20%	100	23.6	11.78	0	19.04	4	14.64	2
20	20%	1000	23.6	5.50	0	18.88	4	17.23	2
20	20%	10000	23.7	1.91	0	19.01	4	18.27	3
				
			Mean	3.72	0.3	10.66	4.7	9.07	3.7
			SD	3.558	0.71	6.586	0.97	6.209	1.66

*Strong*: The alternative hypothesis is strong implying that data come from a Normal(0.97, 1). *N*: sample size. % *effect*: % of real true alternatives. *S*: number of tests. *Significant*: % of significants before adjustment. *Detected*: % of significant tests after adjustment. *FDR*: false discovery rate. SB: Sequential Bonferroni. BH: Benjamini and Hochberg. SGoF: Sequential Goodness of Fit.

**Table 5:** Percentages of significant cases detected (*Detected*) and false discovery rate (*FDR*) after multitest adjustment when the *p*-values come from families of homogeneity tests where some (% effect) of the alternative hypotheses were true.

	*Strong*			SB		BH		SGoF	
				
*N*	*% effect*	*S*	*Significant*	*Detected*	*FDR*	*Detected*	*FDR*	*Detected*	*FDR*
20	5%	100	7.2	0.26	21	0.29	23	0.38	55
20	5%	1000	7.1	0.04	8	0.05	10	0.77	34
20	5%	10000	7.1	0.00	5	0.02	5	1.67	21
20	10%	100	9.0	0.42	13	0.54	17	1.04	32
20	10%	1000	9.0	0.07	3	0.15	7	2.58	20
20	10%	10000	9.0	0.01	2	0.07	2	3.55	12
20	20%	100	12.5	0.77	7	1.27	8	3.62	17
20	20%	1000	12.7	0.13	2	0.65	3	6.29	10
20	20%	10000	12.8	0.02	1	0.47	2	7.33	7
				
			Mean	0.19	6.9	0.39	8.6	3.03	23.1
			SD	0.258	6.51	0.402	7.20	2.45	15.09

40	5%	100	8.4	1.05	7	1.36	12	0.67	26
40	5%	1000	8.5	0.29	2	0.95	6	2.08	14
40	5%	10000	8.5	0.11	1	0.92	2	3.03	6
40	10%	100	11.8	1.98	3	3.12	7	2.90	12
40	10%	1000	11.9	0.58	1	2.76	4	5.50	9
40	10%	10000	11.8	0.21	0	2.83	1	6.42	2
40	20%	100	18.7	3.76	1	8.13	5	9.67	7
40	20%	1000	18.6	1.17	0	7.72	2	12.23	4
40	20%	10000	18.6	0.42	0	7.62	0	13.18	1
				
			Mean	1.06	1.7	3.93	4.3	6.19	9
			SD	1.173	2.24	3.029	3.71	4.557	7.70

*Strong*: The alternative hypothesis is strong.*N*: sample size. % *effect*: % of real true alternatives. *S*: number of tests. *Significant*: % of significants before adjustment. *Detected*: % of significant tests after adjustment. *FDR*: false discovery rate. SB: Sequential Bonferroni. BH: Benjamini and Hochberg. SGoF: Sequential Goodness of Fit.

**Figure 1:**
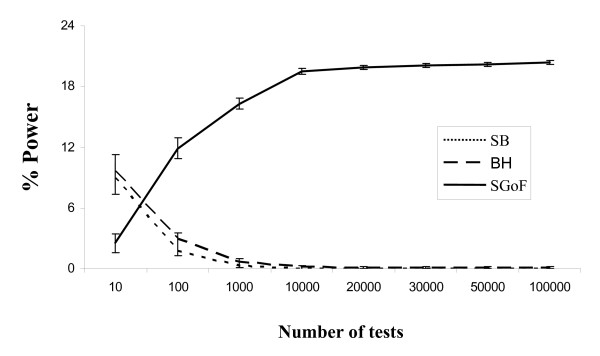
**Power with different number of tests**. Percentage (%) of power for different number of tests. The family of tests was the one-sample t tests with 20% of them coming from a N(0.36, 1) and sample size 20. Values are averages through 1,000 replicates. Error bars represent standard deviations between replicates. The power is defined as the number of true discoveries divided by the total of existing effects (false null hypotheses).

To further study the effect of the number of tests onto the power we performed different sets of one-sample t tests, from 10 to 100,000 tests (Figure 1), with 20% of them coming from the weak alternative. As it can be appreciated in the figure the power of SGoF (defined as the ratiobetween the number of true positives and the number of false null hypotheses -or effects) increases with the number of tests while the power of SB and BH diminishes. This same pattern can be observed for any case in the tables (see Tables 2 to 5 and compare the three *S *rows of each case) for 100, 1000 and 10,000 tests.

**Figure 2:**
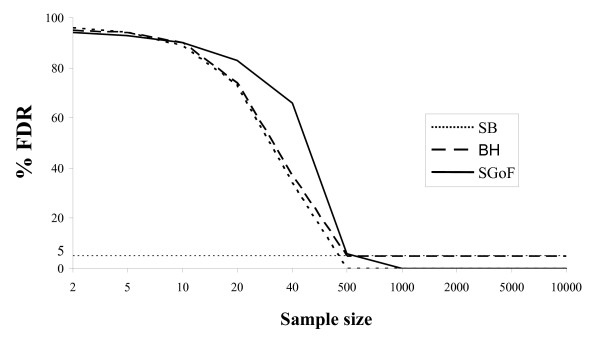
**False discovery rate with different sample sizes**. Percentage (%) of FDR for different sample sizes. The family of tests was 10,000 one-sample t tests with 5% of them coming from a N (0.36, 1). Values are averages through 1,000 replicates.

Concerning the magnitude of the deviation from the null hypothesis, the closer the alternative is to the null, i.e. the weaker the effects (Tables 2 and 3), the higher is the FDR, and vice versa (Tables 4 and 5). Sample size is also critical for controlling FDR. When sample size is small (*N *= 5 for t-tests and *N *= 20 for G-tests) FDR is not being controlled whatever the adjustment used. In the case of SB and BH it seems that the FDR decreases faster than with SGoF but this is simply because under SB or BH there are almost no discoveries so that the margin for false ones is reduced. We further studied how sample size impacts onto FDR control. Therefore, we performed simulations through a wide range of sample sizes, with a family of 10,000 one-sample t-tests with 5% of them coming from the weak alternative and computed the % of false discoveries for the different adjustment methods (Figure 2). It can be appreciated that the effect of sample size is important, indeed with a sample size of 40 the FDR is almost one order of magnitude away from its nominal value under the BH method (5%). Under asymptotic conditions SB and SGoF methods reach null rates and BH reaches the nominal value *q *(0.05). For sample sizes comprised between 20 and 40, SGoF shows higher FDR simply because it is the only one that detects some true discoveries (SB and BH detect an average value lower than 1 through the 1000 replicates, not shown).

In summary, SGoF appears to outperform SB and BH when the effects were weak (Tables 2 and 3) and affected a high proportion of tests (10% or 20%). In fact, BH only behaves slightly better than SGof under the stronger effect and large sample size in the one-sample t tests (*N *= 20 in Table 4). In the case of the G test with the stronger effect and larger sample size, both methods behave similarly with slight advantage for SGoF (see *N *= 40 in Table 5). Therefore, in Tables 2 and 3, mean percentages of discoveries are one order of magnitude larger in SGoF but false discoveries are in the worst cases only twice or three times higher. The standard deviation through replicates [see Additional file 1] is, in general, slightly higher for SGoF. Under the most favorable conditions, SGoF shows statistical power 2 orders of magnitude higher than the others. For example, under the G tests with *S *= 10000, *N *= 40 and 20% of the comparisons,*i.e*. 2000 tests having a true but weak effect, BH identifies 4 ± 1 discoveries while SGoF detects 344 ± 41 (Table 3). Notably both methods give almost the same percentage of false discovery rate (7 and 6%, respectively).

**Figure 3:**
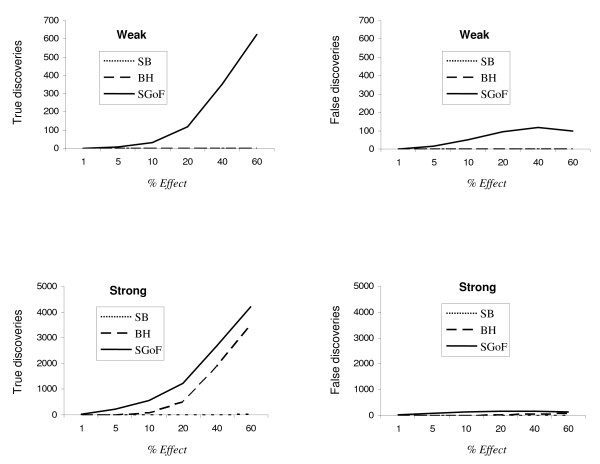
**Comparison of the multitest adjustments for one-sample t tests**. Number of true and false discoveries obtained under the different multitest adjustment methods over a varying proportion (*% Effect*) of the alternative hypothesis contributing to the family of comparisons. The sample size of each one-sample t test was intermediate (*N *= 10). The alternative hypothesis represents *Weak *or *Strong *deviations from the null one. The absolute number of detected true discoveries among 10,000 is shown on the left side, while the absolute number of false discoveries is presented on the right side. Values are averages through 1,000 replicates.

**Figure 4:**
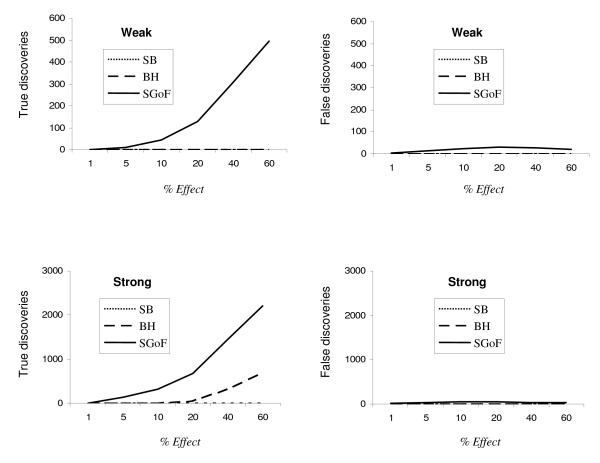
**Comparison of the multitest adjustments for homogeneity tests**. Number of true and false discoveries obtained under the different multitest adjustment methods over a varying proportion (*% Effect*) of the alternative hypothesis contributing to the family of comparisons. The sample size of each homogeneity test was small (*N *= 20). The alternative hypothesis represents *Weak *or *Strong *deviations from the null one. The absolute number of detected true discoveries among 10,000 is shown on the left side, while the absolute number of false discoveries is presented on the right side. Values are averages through 1,000 replicates.

To further study how the efficiency of the different methods depends on the percentage of tests in which the alternative is true (% effect) we simulated, for both kinds of tests, a case with high number of tests, *S *= 10,000, through a wide range of % of effects (Figures 3 and 4). The left panel of both figures shows the absolute number of true discoveries, that is, rejections of the null when is false, for the three multitest adjustments studied. The right panel shows the false discoveries, i.e. rejections of the null when it is true. Noticeably, for the weak alternatives case, only SGoF has power to identify true discoveries. Concerning the SGoF false discoveries, the FDR is higher under the one-sample t test (right panel in Figure 3) than under the G test (right panel in Figure 4), maybe due to the smaller sample size of the first (10 versus 20). As expected from the term (1-λ) in equation (2) the power increases with the percentage of tests having a true effect (Figures 3 and 4, left panel). For the strong alternatives, BH performed only slightly worse than SGoF in the case of one-sample t tests, although SGoF performed still better in the case of G tests.

### Example of application

Martínez-Fernández et al. [24] accomplished a proteomic study in which they performed 1498 tests. Of these, 21 were statistically significant (using a significance level of 0.2%). After correction with the BH method they did not get any significant case. However, using a G-test they rejected at a 0.2% significance level that the 21 significant tests could be explained by chance. If we apply the SGoF test on this data set (with α = 0.002), we find 12 significants. Notice that the same significance level was used in the family of tests and in the two multitest methods discussed above. Thus, all the 12 extra significant cases could be hardly considered as type I errors, suggesting that they could be considered candidate genes for future detailed biochemical studies.

### Implementation of the method

We provide a computer program which allows to obtain the multitest adjustment probability methods used in this work (B, SB, BH and SGoF;[25]). SGoF is calculated by an exact binomial test when the number of tests is lower than 100 and by a G test with the Williams' correction in any other case. In addition, a more conservative SGoF adjustment using the Yate's correction is also given.

## Discussion

The use of controlling FDR based methods for multitest adjustment has implied an obvious improvement by increasing the statistical power in families of comparisons [26]. However, such an improvement is far from being useful for experimentalists under all the circumstances, in particular when a relatively small sample size and a high number of comparisons are involved. In such conditions, classical multitest adjustments are known to have low statistical power [16]. In fact, a great number of controlling FDR based methods have been proposed trying to further improve its applicability although with moderate results [11,14-17]. Here we suggest a completely different approach, by using a sequential goodness of fit on the set of comparisons, which may help in some of the circumstances where the FDR-based approaches fail to find true discoveries. Similar to Bonferroni techniques, the SGoF metatest controls for FWER (FamilyWise Error Rate). Given a number *S *of tests, in Bonferroni technique the error rate per comparison is fixed to α/S. Therefore, this value diminishes as the number *S *of tests is higher. The problem is that the power to detect true discoveries also depends on this error rate. With a very stringent significance level we will have very low power. Importantly, in the case of SGoF, the per test error rate is proportional to α/*S *by a factor that increases with the number of tests resolving in this way the trade-off between type I error and statistical power. Therefore, the power increases with the number of tests though the family wise error rate is being controlled to avoid a high false discovery rate. As far as we know, there are not other multitest adjustment methods with this desirable property of increasing power with the number of tests. However, as can be expected from equation (2) and can be seen in Figure 1 such increase is not lineal. Therefore, it could be suggested that increasing the number of tests up to 1000 or 10,000 will increase considerably the statistical power of the SGoF adjustment, but above 10,000 the increase will become slighter (Figure 1). This suggests that using more than 10,000 comparisons could not offer a clear advantage.

Another issue concerning the statistical power of SGoF seems to be the percentage of tests in which the alternative is true (% effect) which has a clear impact onto the discoveries rates. In the case of SB and BH this impact is more difficult to follow from the tables because there is a trade-off with the increasing number of tests (which reduces the power). However with SGoF the effect is very clear because, as is expected from equation (2), both the % of effects (1-λ) and the number *S *of tests increase the power.

Obviously, because SGoF does not perform so stringent control neither on the per test error rate nor in the FDR, this implies that FDR is being allowed to be higher than with SB and BH methods. However, given a number *K *of observed significants, as power increases, FDR is expected to diminish because in such a case the proportion of true discoveries approaches 1. Therefore, SGoF will attain an indirect control of FDR with large numbers of tests and/or effects involved. That is, SGoF will behave especially well compared to the classical methods when the alternative is weak and both the number of effects through the family of tests and the number of tests involved are high. In this case, SGoF can be up to two orders of magnitude more powerful than the other methods, maintaining at the same time acceptable FDR values.

We have also observed that if the *p*-values are not correctly calculated the FDR will be uncontrolled as occurs with any other multitest adjustment method. This is noteworthy because empirical studies do not usually involve large sample sizes within each test. Known multitest adjustment methods can have very good asymptotic statistical properties. In fact, both SB and BH have very good power with the kind of tests assayed when the sample size is as large as 500 (not shown). The problem is that empirical science does not work on the asymptotic arena but on finite sample size. As we have seen, the assumption of controlled FDR fails when sample size is small, at least under one-sample t and homogeneity G-tests. Additionally, the classical adjustment methods (B, SB and BH) have low power when the number of tests is high and/or the effects are weak. Indeed in these conditions, SGoF should be considered as an interesting method to detect that some kind of true effect exists though we are not confident in that all detected positives are true discoveries. In addition, some uncertainty exists when significant probabilities have exactly the same values. For example, if 9 out of 10 comparisons have a p-value below 0.05, say 0.049, SGoF will show that 8 can not be explained by chance, but the researcher has no way to choose among the 9. On the other side alternative multitest adjustment methods (BH or others) cannot find any significant case. Nevertheless, from an experimentalist point of view, it will be more useful to know that at least 8 hypotheses deserve more detailed studies than to just ignore all of them. In cases like this, under the SGoF method, the 8 significant tests will be chosen randomly from the 9 available.

Concerning statistical properties as conservativeness, sensitivity and specificity we have computed the degree of conservativeness [27] and performed ROC analysis [28] for the same cases as in Figure 3 [see Additional file 2]. The results just confirm the good properties of SGoF as already expected from the higher per comparison error rates (see property 2) and the true and false discoveries numbers (see Figure 3).

Another important topic concerning multiple hypothesis testing efforts applied to high-throughput experiments is the intrinsic inter-dependency in gene effects. We would like to note that correlation can have important effect onto FDR-based adjustment methods [29]. However, it is usually considered a kind of dependence in gene effects called weak-dependence which corresponds to local effects between a small number of genes [30]. It has been shown that under the assumption of the so-called weak-dependence, the FDR-based methods are still useful provided that the number of tests is large enough [20,30]. SGoF does not consider the p-values individually but the proportion of significant ones and this should make it more robust to dependence issues. Therefore, we expect at least the same or better performance for SGoF than for FDR-based methods when considering gene dependencies. Our preliminary results (not shown) indicate that dependence has no effect onto SGoF power provided that the blocks with correlated genes are small. Indeed with blocks as large as 100 genes and correlation as high as 0.9 the loss in power is small. Furthermore, short blocks of correlated genes is what is expected in genome and proteome wide studies [20,29]. Additionally, we have observed that if the blocks are short the magnitude of the correlation has a minor effect. Nevertheless, we think that such topic deserves further study.

Finally, we note that we have obtained *p*-values via simulation from two kind of tests, one-sample t-test which is widely used, and also via homogeneity tests, that are also frequently involved in multiple comparisons [31]. In addition, SGoF should be of general utility under other families of multiple comparisons, although this should deserve further investigation. The failure of classical multitest adjustments to deal with a huge number of tests (>1000) has been considered as a key problem in many omic technologies [16], and so SGoF comes to contribute to a well-known need.

## Conclusion

We propose a new multitest adjustment, based on a sequential goodness of fit metatest (SGoF) which, contrary to other methods, increases its statistical power with the number of tests. Therefore, SGoF should become an interesting strategy for multitest adjustment when working with high-dimensional biological data. The SGoF metatest, jointly with B, SB and BH multitest adjustments, can be easily computed with the software provided.

## Methods

### Algorithm for the Sequential Goodness of Fit (SGoF) metatest

Given a set of *S *independent tests, performed each at a given significance level α, we expect a number (*F *= *S *× α) of false discoveries. Let *K *be the observed number of cases with *p*-value below the threshold α. The SGoF algorithm works as follows:

1) Input: A list of *S *sorted *p*-values, from minor to major, (note that in the program that we provide this is not necessary because the program itself performs the sorting).

2) Set *R *= *K*, the number of *p*-values below the threshold (α).

3) Repeat: Test (binomial or chi-square) if the *R *observed discoveries deviate significantly from the expected *F *ones.

a. If the test is significant: count a new significant (corresponding to the smallest *p*-value), then update the list of observed *p*-values, i.e. decrease in one unit the number *R *and consequently increase in one unit the number of values above the threshold (to hold *S *constant). Repeat the process from 3).

b. If the test is not significant: stop the process and go to 4)

4) The output of the program is the number of significants detected in step 3)

This metatest is an exact binomial test. However, when the number of cases is large (*S *> 100) it can be approximated by a chi-square or a G test obtaining exactly identical results.

### Generation of families of *p*-values by simulation

In order to compare the efficiency of the proposed SGoF metatest, we need to generate a variable number of comparisons with different known (*a priori*) probabilities of true discoveries. To generate a list of *p*-values we performed two different kinds of tests, namely, t and homogeneity tests. Whatever the kind of test, two different scenarios were assayed and, for each, three different numbers of experiments, *S*, were simulated, 100, 1000 and 10000. The scenarios were:

1) The null model is always true (intersection null hypothesis).

2) An alternative model is true in some of the *S *tests. We assayed three different percentages (% effect = 5, 10 and 20%) for the alternative model being true with respect to the total number *S *of tests.

Therefore, there were a total of 123 different cases from the two kinds of tests and all combinations of sample sizes (see below), number of tests, % of effect and alternative models. Each test case was replicated 1000 times to provide empirical standard deviations in the estimates of multiple adjustments.

#### t tests

To perform the series of *t *tests we implemented a modification of the procedure outlined in Brown and Rusell [32]. First, we got standard normal deviates, *x*, that, after a t-test, were transformed to *p*-values via the incomplete beta function [33]. The mean of the normal deviates generated for the null hypothesis was 0. We chose the mean for the alternative hypothesis so that the probability of a *p*-value less than 5% should be either 0.10 or 0.25 under asymptotic conditions. This means an effect of 0.36 i.e. sampling from N(0.36,1) or an effect of 0.97 i.e. sampling from N(0.97,1), respectively. As explained above we assayed three different percentages (% effect = 5, 10 and 20%) for the alternative model being true with respect to the total number *S *of tests. We generated the normal deviates under a given, null or alternative, distribution, in blocks of sample size *N *= 5, 10 or 20. Because we performed a two-tailed t test with *N*-1 degrees of freedom, at the 5% significance level, there was a power of 0.10, 0.18 and 0.33 with sample sizes 5, 10 and 20 respectively when we tested versus the alternative with mean 0.36, and a power of 0.38, 0.78 and 0.98, respectively, for the alternative with mean 0.97. These were, at each test, the probabilities for rejecting the null being false.

#### G tests

We simulated a homogeneity test comparing the frequencies of two classes (A and B) in two populations (1 and 2). As above, two different situations, the null and the alternative model, were simulated. In the latter, the effect could be weak or strong (Table 6). To simulate the null model we resampled data from a 2 × 2 table with equal expected probabilities of allocating data in cells (see Table 6, null case) until a particular sample size (*N*) was achieved. Two different sample sizes were used, namely, 20 and 40. For each sample size, the process was repeated until a collection *S *(number of tests) of independent tables was obtained.

**Table 6:** Probabilities used in the simulation to resample the null and the two alternative hypotheses (weak and strong deviations from the null hypothesis) in a 2 × 2 homogeneity test.

	Null		Weak		Strong	
			
	A	B	A	B	A	B
	
Pop 1	1	1	1	0.67	1	0.43
Pop 2	1	1	0.67	1	0.43	1

On each re-sampled table, we applied a goodness of fit homogeneity test, which follows a chi-square distribution with one degree of freedom [1]. In such a homogeneity test, the expected numbers per cell were obtained from frequencies of classes (A/B) and populations (1/2) under the null hypothesis of homogeneity. In order to obtain a simulated empirical rate of false positives as close as possible to the level of significance used, we applied the Williams' correction. Additionally, when sample size was 20 we also used the Yates correction [1].

To simulate the alternative hypothesis, a percentage of the simulated tables (*% effect *5%, 10% and 20%) were re-sampled from an alternative model which could represent *weak *or *strong *deviations from the null case (see Table 6). For example, when simulating 100 tables (*S *= 100) with a percentage of 5% of weak effects, that means that the simulation generated 95 tables from the null model and 5 from the weak alternative model from Table 6.

Notice that, both for t and G tests, we knew *a priori *which *p*-value came from a null or an alternative model; therefore, after multitest adjustment we could check *a posteriori *which of the significant tests (discoveries) were false (the false discovery rate, FDR) and which were true. Thus, when necessary, the power of a given multitest adjustment was measured as the number of true discoveries divided by the total of existing effects (false null hypotheses) and the FDR was measured as the number of false discoveries divided by the total discoveries.

### Comparing efficiencies of alternative multiple adjustments

From the collection of *p*-values available from each simulated case we applied some of the most common multitest corrections. The Bonferroni correction (B), adjusts the level of significance by dividing it by the number of tests used [2]. The Sequential Bonferroni adjustment (SB) divides the level of significance by the number of tests, sequentially subtracting those which were previously significant [2,34]. In this manner, it allows for controlling the familywise error rate (FWER). The false discovery rate (FDR) adjustment was described by Benjamini and Hochberg [3] and we used its simplest version, that we refer as to BH, which first ranks all probability values and second, verifies if



*p*(*i*) being the probability of the significant test in rank *i*, *S *the number of tests and *q *the level of significance chosen (so that the FDR is maintained below *q*, provided that some conditions regarding the distribution of the *p*-values hold [35]). Let *k *be the largest *i *for which *p*(*i*) 7#8804; (*i*/*S*) × *q *and then declare the hypothesis corresponding to the smallest *k p*-values as significant. Since controlling for FDR is less stringent than for FWER, the FDR based procedures can exhibit higher power in certain conditions. Unless otherwise stated, we always used *q *= α = 0.05.

Finally, we also applied the sequential goodness of fit metatest (SGoF) described above to perform multitest correction. Therefore, we tested which of the observed significant cases could not be explained by chance following the 5% significance level. This goodness-of-fit metatest was calculated by a G-test using the William's correction as we always simulated 100 or more tests, approximating a chi-squared distribution with one degree of freedom. Additionally, we also performed the whole set of simulations using the exact binomial test, and the results were identical with *S *≥ 1000, though slightly better for *S *= 100 (results not shown) as expected because with large sample sizes the G test with one degree of freedom approaches very well to the binomial test [1].

## Authors' contributions

E R-A had the original idea for the SGoF method. AC-R implemented the simulations and the SGoF program. JU-A worked out the statistical properties. The three authors designed the final algorithm and wrote the manuscript. All authors read and approved the final manuscript.

## Supplementary Material

Additional file 1 **Additional tables**. The data provided include the standard deviations between replicates corresponding to data from tables 2 to 5 of the manuscript. Click here for file

Additional file 2 **Additional figures**. Degree of conservativeness (Figure S1) and ROC analysis (Figure S2) for the same cases as in Figure 3. Click here for file
