# Supplementary material for: A new multitest correction (SGoF) that increases its statistical power when increasing the number of tests
Source: BMC Bioinformatics. 2009 Jul 8;10:209. doi: 10.1186/1471-2105-10-209 (PMC2719628; doi:10.1186/1471-2105-10-209)
Supplement: Additional file 1 — Additional tables. The data provided include the standard deviations between replicates corresponding to data from tables 2 to 5 of the manuscript. [file 1471-2105-10-209-S1.doc]

# A new multitest correction (SGoF) that increases its statistical power when increasing the number of tests

A. Carvajal-Rodríguez^1§^, J. de Uña-Alvarez^2^ & E. Rolán-Alvarez^1^

**Additional Tables**

TABLE ST1: Standard deviations (SD) from data presented in Table 2 in the manuscript when the *p*-values come from families of one-sample t tests where some (% effect) of the alternative hypotheses were true.

|  | *Weak* |  |  | SB |  | BH |  | SGoF |
| --- | --- | --- | --- | --- | --- | --- | --- | --- |
| *N* | *% effect* | *S* | *Significant* | *SD* |  | *SD* |  | *SD* |
| 5 | 5% | 100 | 5.2 | 0.01 |  | 0.02 |  | 0.05 |
| 5 | 5% | 1000 | 5.2 | 0 |  | 0 |  | 0.03 |
| 5 | 5% | 10000 | 5.2 | 0 |  | 0 |  | 0.05 |
| 5 | 10% | 100 | 5.4 | 0.02 |  | 0.02 |  | 0.07 |
| 5 | 10% | 1000 | 5.5 | 0 |  | 0 |  | 0.06 |
| 5 | 10% | 10000 | 5.5 | 0 |  | 0 |  | 0.14 |
| 5 | 20% | 100 | 5.9 | 0.02 |  | 0.02 |  | 0.07 |
| 5 | 20% | 1000 | 6.0 | 0 |  | 0 |  | 0.11 |
| 5 | 20% | 10000 | 6.0 | 0 |  | 0 |  | 0.29 |
|  |  |  |  |  |  |  |  |  |
| 10 | 5% | 100 | 5.7 | 0.02 |  | 0.03 |  | 0.04 |
| 10 | 5% | 1000 | 5.6 | 0 |  | 0 |  | 0.06 |
| 10 | 5% | 10000 | 5.6 | 0 |  | 0 |  | 0.18 |
| 10 | 10% | 100 | 6.2 | 0.02 |  | 0.03 |  | 0.15 |
| 10 | 10% | 1000 | 6.2 | 0 |  | 0 |  | 0.12 |
| 10 | 10% | 10000 | 6.3 | 0 |  | 0 |  | 0.29 |
| 10 | 20% | 100 | 7.6 | 0.02 |  | 0.04 |  | 0.15 |
| 10 | 20% | 1000 | 7.5 | 0 |  | 0.01 |  | 0.31 |
| 10 | 20% | 10000 | 7.5 | 0 |  | 0 |  | 0.37 |
|  |  |  |  |  |  |  |  |  |
| 20 | 5% | 100 | 6.4 | 0.03 |  | 0.05 |  | 0.09 |
| 20 | 5% | 1000 | 6.5 | 0 |  | 0.01 |  | 0.2 |
| 20 | 5% | 10000 | 6.4 | 0 |  | 0 |  | 0.31 |
| 20 | 10% | 100 | 7.8 | 0.03 |  | 0.06 |  | 0.25 |
| 20 | 10% | 1000 | 7.9 | 0 |  | 0.02 |  | 0.38 |
| 20 | 10% | 10000 | 7.9 | 0 |  | 0 |  | 0.38 |
| 20 | 20% | 100 | 10.7 | 0.04 |  | 0.14 |  | 0.47 |
| 20 | 20% | 1000 | 10.8 | 0.01 |  | 0.05 |  | 0.51 |
| 20 | 20% | 10000 | 10.8 | 0 |  | 0.02 |  | 0.6 |
|  |  |  |  |  |  |  |  |  |

*Weak*: The alternative hypothesis is weak implying that data come from a Normal(0.36, 1). *N* : sample size. % *effect*: % of real true alternatives. *S*: number of tests. *Significant*: % of significants before adjustment. SB: Sequential Bonferroni. BH: Benjamini and Hochberg. SGoF: Sequential Goodness of Fit.

TABLE ST2: Standard deviations (SD) from data presented in Table 3 in the manuscript when the *p*-values come from families of homogeneity tests where some (% effect) of the alternative hypotheses were true.

|  | *Weak* |  |  | SB |  | BH |  | SGoF |
| --- | --- | --- | --- | --- | --- | --- | --- | --- |
| *N* | *% effect* | *S* | *Significant* | *SD* |  | *SD* |  | *SD* |
| 20 | 5% | 100 | 5.7 | 0.02 |  | 0.02 |  | 0.05 |
| 20 | 5% | 1000 | 5.7 | 0 |  | 0 |  | 0.06 |
| 20 | 5% | 10000 | 5.7 | 0 |  | 0 |  | 0.23 |
| 20 | 10% | 100 | 6.2 | 0.02 |  | 0.02 |  | 0.04 |
| 20 | 10% | 1000 | 6.1 | 0 |  | 0 |  | 0.11 |
| 20 | 10% | 10000 | 6.1 | 0 |  | 0 |  | 0.3 |
| 20 | 20% | 100 | 6.8 | 0.03 |  | 0.04 |  | 0.1 |
| 20 | 20% | 1000 | 7.0 | 0 |  | 0 |  | 0.26 |
| 20 | 20% | 10000 | 7.0 | 0 |  | 0 |  | 0.29 |
|  |  |  |  |  |  |  |  |  |
| 40 | 5% | 100 | 6.0 | 0.03 |  | 0.04 |  | 0.08 |
| 40 | 5% | 1000 | 6.0 | 0 |  | 0.01 |  | 0.1 |
| 40 | 5% | 10000 | 6.0 | 0 |  | 0 |  | 0.3 |
| 40 | 10% | 100 | 7.0 | 0.03 |  | 0.05 |  | 0.12 |
| 40 | 10% | 1000 | 7.0 | 0 |  | 0.01 |  | 0.23 |
| 40 | 10% | 10000 | 7.0 | 0 |  | 0 |  | 0.34 |
| 40 | 20% | 100 | 8.9 | 0.05 |  | 0.1 |  | 0.26 |
| 40 | 20% | 1000 | 8.9 | 0.01 |  | 0.03 |  | 0.43 |
| 40 | 20% | 10000 | 8.9 | 0 |  | 0.01 |  | 0.41 |
|  |  |  |  |  |  |  |  |  |

Weak: *Weak*: The alternative hypothesis is weak. *N* : sample size. % *effect*: % of real true alternatives. *S*: number of tests. *Significant*: % of significants before adjustment. SB: Sequential Bonferroni. BH: Benjamini and Hochberg. SGoF: Sequential Goodness of Fit.

TABLE ST3: Standard deviations (SD) from data presented in Table 4 in the manuscript when the *p*-values come from families of one-sample t tests where some (% effect) of the alternative hypotheses were true.

|  | *Strong* |  |  | SB |  | BH |  | SGoF |
| --- | --- | --- | --- | --- | --- | --- | --- | --- |
| *N* | *% effect* | *S* | *Significant* | *SD* |  | *SD* |  | *SD* |
| 5 | 5% | 100 | 6.6 | 0.02 |  | 0.03 |  | 0.12 |
| 5 | 5% | 1000 | 6.7 | 0 |  | 0 |  | 0.24 |
| 5 | 5% | 10000 | 6.7 | 0 |  | 0 |  | 0.36 |
| 5 | 10% | 100 | 8.5 | 0.03 |  | 0.04 |  | 0.24 |
| 5 | 10% | 1000 | 8.4 | 0 |  | 0 |  | 0.44 |
| 5 | 10% | 10000 | 8.3 | 0 |  | 0 |  | 0.41 |
| 5 | 20% | 100 | 11.7 | 0.03 |  | 0.08 |  | 0.4 |
| 5 | 20% | 1000 | 11.7 | 0 |  | 0.01 |  | 0.55 |
| 5 | 20% | 10000 | 11.7 | 0 |  | 0 |  | 0.55 |
|  |  |  |  |  |  |  |  |  |
| 10 | 5% | 100 | 8.7 | 0.05 |  | 0.15 |  | 0.29 |
| 10 | 5% | 1000 | 8.6 | 0.01 |  | 0.07 |  | 0.45 |
| 10 | 5% | 10000 | 8.6 | 0 |  | 0.06 |  | 0.45 |
| 10 | 10% | 100 | 12.3 | 0.07 |  | 0.35 |  | 0.48 |
| 10 | 10% | 1000 | 12.3 | 0.01 |  | 0.33 |  | 0.65 |
| 10 | 10% | 10000 | 12.3 | 0 |  | 0.42 |  | 0.64 |
| 10 | 20% | 100 | 19.5 | 0.15 |  | 1.04 |  | 0.92 |
| 10 | 20% | 1000 | 19.6 | 0.02 |  | 1.28 |  | 0.81 |
| 10 | 20% | 10000 | 19.6 | 0 |  | 1.47 |  | 0.85 |
|  |  |  |  |  |  |  |  |  |
| 20 | 5% | 100 | 9.8 | 0.17 |  | 0.31 |  | 0.3 |
| 20 | 5% | 1000 | 9.7 | 0.07 |  | 0.32 |  | 0.44 |
| 20 | 5% | 10000 | 9.7 | 0.03 |  | 0.33 |  | 0.47 |
| 20 | 10% | 100 | 14.4 | 0.37 |  | 0.64 |  | 0.61 |
| 20 | 10% | 1000 | 14.3 | 0.14 |  | 0.62 |  | 0.66 |
| 20 | 10% | 10000 | 14.3 | 0.05 |  | 0.64 |  | 0.67 |
| 20 | 20% | 100 | 23.6 | 0.57 |  | 1.06 |  | 1 |
| 20 | 20% | 1000 | 23.6 | 0.28 |  | 1.06 |  | 0.96 |
| 20 | 20% | 10000 | 23.7 | 0.1 |  | 1.01 |  | 0.95 |
|  |  |  |  |  |  |  |  |  |

Strong: The alternative hypothesis is strong implying that data come from a N(0.97, 1). *N* : sample size. % *effect*: % of real true alternatives. *S*: number of tests. *Significant*: % of significants before adjustment. SB: Sequential Bonferroni. BH: Benjamini and Hochberg. SGoF: Sequential Goodness of Fit.

TABLE ST4: Standard deviations (SD) from data presented in Table 5 in the manuscript when the *p*-values come from families of homogeneity tests where some (% effect) of the alternative hypotheses were true.

|  | *Strong* |  |  | SB |  | BH |  | SGoF |
| --- | --- | --- | --- | --- | --- | --- | --- | --- |
| *N* | *% effect* | *S* | *Significant* | *SD* |  | *SD* |  | *SD* |
| 20 | 5% | 100 | 7.2 | 0.03 |  | 0.06 |  | 0.1 |
| 20 | 5% | 1000 | 7.1 | 0 |  | 0.01 |  | 0.23 |
| 20 | 5% | 10000 | 7.1 | 0 |  | 0 |  | 0.32 |
| 20 | 10% | 100 | 9.0 | 0.05 |  | 0.12 |  | 0.21 |
| 20 | 10% | 1000 | 9.0 | 0.01 |  | 0.04 |  | 0.38 |
| 20 | 10% | 10000 | 9.0 | 0 |  | 0.02 |  | 0.39 |
| 20 | 20% | 100 | 12.5 | 0.07 |  | 0.24 |  | 0.4 |
| 20 | 20% | 1000 | 12.7 | 0.01 |  | 0.13 |  | 0.5 |
| 20 | 20% | 10000 | 12.8 | 0 |  | 0.3 |  | 0.48 |
|  |  |  |  |  |  |  |  |  |
| 40 | 5% | 100 | 8.4 | 0.06 |  | 0.12 |  | 0.14 |
| 40 | 5% | 1000 | 8.5 | 0.02 |  | 0.11 |  | 0.32 |
| 40 | 5% | 10000 | 8.5 | 0.01 |  | 0.2 |  | 0.31 |
| 40 | 10% | 100 | 11.8 | 0.1 |  | 0.25 |  | 0.31 |
| 40 | 10% | 1000 | 11.9 | 0.03 |  | 0.32 |  | 0.34 |
| 40 | 10% | 10000 | 11.8 | 0.01 |  | 0.77 |  | 0.37 |
| 40 | 20% | 100 | 18.7 | 0.18 |  | 0.58 |  | 0.42 |
| 40 | 20% | 1000 | 18.6 | 0.06 |  | 0.91 |  | 0.43 |
| 40 | 20% | 10000 | 18.6 | 0.02 |  | 2.04 |  | 0.39 |
|  |  |  |  |  |  |  |  |  |

Strong: The alternative hypothesis is strong. *N* : sample size. % *effect*: % of real true alternatives. *S*: number of tests. *Significant*: % of significants before adjustment. SB: Sequential Bonferroni. BH: Benjamini and Hochberg. SGoF: Sequential Goodness of Fit.
