# Supplementary material for: A new multitest correction (SGoF) that increases its statistical power when increasing the number of tests
Source: BMC Bioinformatics. 2009 Jul 8;10:209. doi: 10.1186/1471-2105-10-209 (PMC2719628; doi:10.1186/1471-2105-10-209)
Supplement: Additional file 2 — Additional figures. Degree of conservativeness (Figure S1) and ROC analysis (Figure S2) for the same cases as in Figure 3. [file 1471-2105-10-209-S2.doc]

**Additional Figures**

## Figure S1 – Conservativeness degree of the multitest adjustments for one-sample t tests

Conservativeness degree for the findings CD normalized by the average number of discoveries *R* represented over a varying proportion (*% Effect*) of the alternative hypothesis contributing to the family of comparisons. Given that having *R* discoveries we expect αR of them being false ones, the CD is computed as the number of times among 10,000 tests in which false discoveries were less than the expected. If the average number of discoveries *R* is minor than one, the normalized CD is set to a maximum of 1 (CD/R is not allowed to be higher than 1). The sample size of each one-sample t test was intermediate (*N* = 10). The alternative hypothesis represents *Weak* or *Strong* deviations from the null one. Values are averages through 100 runs. SB: Sequential Bonferroni. BH: Benjamini and Hochberg. SGoF: Sequential Goodness of Fit.

## Figure S2 – ROC curves of the multitest adjustments for one-sample t tests

Sensitivity (Rp), i.e. proportion of true effects which are correctly identified as such, plotted against 1 – specificity, (Rf), i.e. 1 - proportion of nulls which are correctly identified, for different proportion (*% Effect* from 1 to 60%) of the alternative hypothesis contributing to the family of comparisons. The sample size of each one-sample t test was intermediate (*N* = 10). The alternative hypothesis represents *Weak* or *Strong* deviations from the null one. Values are averages through 100 runs. NDL: no-discrimination line. SB: Sequential Bonferroni. BH: Benjamini and Hochberg. SGoF: Sequential Goodness of Fit. If a given legend and line do not appear, as SB and BH in the weak case, is because in such cases there were not discoveries (true or false) at all.

NDL

SGoF

**Weak**

**Strong**

%Rf

%Rf

%Rp

%Rp

NDL

BH

SGoF
